# The acid tolerance response and pH adaptation of *Enterococcus faecalis* in extract of lime *Citrus aurantiifolia* from Aceh Indonesia

**DOI:** 10.12688/f1000research.13990.2

**Published:** 2018-04-11

**Authors:** Zaki Mubarak, Cut Soraya

**Affiliations:** 1 Faculty of Dentistry, University of Syiah Kuala, Banda Aceh, Indonesia

**Keywords:** Enterococcus faecalis, Lime (Citrus aurantiifolia) extract, acid tolerance, pH adaptation

## Abstract

**Background:** The objective of the present study was to evaluate the acid tolerance response and pH adaptation when *Enterococcus* *faecalis* interacted with extract of lime ( *Citrus aurant* *iifolia*).

**Methods** **:** We used *E. faecalis* ATCC 29212 and lime extract from Aceh, Indonesia. The microbe was analyzed for its pH adaptation, acid tolerance response, and adhesion assay using a light microscope with a magnification of x1000. Further, statistical tests were performed to analyze both correlation and significance of the acid tolerance and pH adaptation as well as the interaction activity.

**Results** **:** *E. faecalis* was able to adapt to a very acidic environment (pH 2.9), which was characterized by an increase in its pH (reaching 4.2) at all concentrations of the lime extract (p < 0.05). *E. faecalis* was also able to provide acid tolerance response to lime extract based on spectrophotometric data (595 nm) (p < 0.05). Also, the interaction activity of *E. faecalis* in different concentrations of lime extract was relatively stable within 6 up to 12 hours (p < 0.05), but it became unstable within 24–72 hours (p > 0.05) based on the mass profiles of its interaction activity.

**Conclusions** **:** *E. faecalis* can adapt to acidic environments (pH 2.9–4.2); it is also able to tolerate acid generated by *Citrus auranti* *ifolia* extract, revealing a stable interaction in the first 6–12 hours.

## Introduction

*Enterococcus faecalis* is a significant agent in the pathogenesis of root canal infections, especially in post-endodontic treatment, with a prevalence of 24–77% in these infections ^1^. This bacterium is very difficult to eliminate because the pathogen can survive in poor nutrient conditions. It can adapt to acidic conditions, including living in the dentin tubule of a closed root canal with a smear layer. It can also express the dominant biofilm protein to maintain its attachment to host cells ^2^.

*E. faecalis* has been shown tolerate to acidic environments as well as to adapt to pH changes, which are the essential virulence factors in maintaining antibacterial balance ^3^. Fisher reported that *E. faecalis* could survive in environments with high NaCl concentrations at extreme temperatures of 5–65°C with a pH of 4.5–10.0 ^4^. Stuart *et al.* ^1^ reported that *E. faecalis* are less sensitive, with a pH of 5.0 at 25°C after it has been incubated for 10 h. The author also found that it has an excellent growth capability at pH 8.5 and low adhesion at pH 7.1 in a medium coated with bovine serum albumin (BSA).

*E. faecalis* is resistant to medication materials such as calcium hydroxide ^5^ and chlorhexidine (CHX) ^6^. The long-term use of both medication materials can lead to parachloroaniline (PAC), causing blockage of the dentinal tubules and eventually becoming toxic ^7^. Fosfomycin may also interfere with acid tolerance systems and pH changes of *E. faecalis* in tooth root canals by inhibiting phosphoenolpyruvate synthetase ^8^.

Indonesia, especially in Aceh has a tropical climate with a variety of plants that can be utilized in medical treatment, including lime ( *Citrus aurantiifolia*). Lime peel extract contains phenols, flavonoids, hydrogen peroxide, tannins, alkaloids, and saponins that have antibacterial, antioxidant, antifungal, analgesic, and anti-inflammatory properties ^9^. Nwankwo ^10^ reported that lime extract helped to prevent *Klebsiella pneumonia*, *Salmonella,* and *Escherichia coli*. Here, the acid in lime extract influenced the bacterial development and cell metabolism. The present study evaluates the acid tolerance response and pH adaptation of *E. faecalis* when the bacterium grows as biofilm in the presence of lime extract with different concentration.

## Methods

### Materials

The lime extract and *E. faecalis* (ATCC-29212) were used in this study. The extractions were conducted at the Laboratory of Microbiology at the Faculty of Veterinary, University of Syiah Kuala, Darussalam, Banda Aceh, Indonesia. The material and bacterium were prepared *in vitro* to analyze the pH adaptation, acid tolerance response, and interaction activity of *E. faecalis* in different concentration of lime extracts.

### Lime extraction

Lime peel was separated from the flesh then dried using dehydrator until the water content reduced to 10%. Dried lime peel was grinded into powders. The powder was put into a glass container and macerated with ethanol 70% for two days and then strained using a gauze. Filtrate was evaporated using a rotary evaporator at 80°C to obtain the pure lime extracts.

### Culture of *E. faecalis*

One colony of *E. faecalis* bacteria was subsequently re-cultured in 5 ml of Mueller-Hinton Broth (MHB) medium (Thermo Fisher Scientific Inc, Paisley, UK) in anaerobic conditions at a temperature of 37°C for 48 hours. Afterward, the *E. faecalis* grown on the liquid medium was synchronized further with McFarland 0.5 (1 × 10 ^8^ CFU/ml) (TM50, Dalynn Biological Inc., Calgary, Canada). The accurate density of McFarland standard was checked using a spectrophotometer with an absorbance reading of 0.08 to 0.1 at 625 nm ^11^.

### Adaptation to pH assay

A total of 50 ml of lime extracts in several different concentrations (100% v/v, 75% v/v, 50% v/v, 25% v/v, 12.5% v/v, and 6.25% v/v) was put into different beaker glasses. Then, 5 ml of *E. faecalis* in MHB (1:10) were added to each of the beakers. The initial pH of each mixture prior to incubation (0 hours) was 2.89, 2.75, 2.91, 2.92, 2.95, 2.98 and 3.10, respectively. Next, bacterium containing beaker was put into incubator (37°C) for 6 hours, 12 hours, 24 hours, 48 hours, and 72 hours in an anaerobic atmosphere using Anaerogen TM GasPack (Oxoid, Basing stoke UK), at each of these times, the beakers’ pH was measured using a pH meter (Thermo Fisher Scientific Inc, Paisley, UK). Various changes in pH from 0 hours to the specified time can be used as an indicator of whether *E. faecalis* has a tolerance response to the acidic environment and can adapt to changing pH ^12^.

### Acid tolerance assay

The cultures of the pH measurements were used to measure the acid tolerance response of *E. faecalis* to lime extract utilizing the principle of spectrophotometry ^13^. The analysis was performed based on the incubation time that had been determined following the measurement of pH shaken at 500 rpm. Here, 50 ul of MHB was put into a microplate in triplicate (Thermo Fisher Scientific Inc., Paisley, UK). The microplate was incubated at room temperature for 15 minutes and vacuumed. The materials tested and *E. faecalis* derived from the incubation processes at 6 h, 12 h, 24 h, 48 h, and 72 hours were added to each well. The microplate was put into an incubator 37 °C for 15 minutes. Then bio-tolerant activity was measured by Elisa Reader (Bio-Rad Laboratories, Hercules, CA) at a wavelength of 595 nm.

### Adhesion assay

Adhesion assay was conducted based on the principles of Gram-staining ^14^. This incubation time-based interaction activity on the microplate 96 wells series was done following the Gamble’s working principle ^15^. It was modified using violet crystalline and safranin staining to confirm the possible occurrence of bacterial contaminants. First, microplate in triplicate wells were coated with 50 μl of MHB (Thermo Fisher Scientific Inc., Paisley, UK) for 15 min, and aspirated. Second, 50 uL of *E. faecalis* in MHB were added and incubated for 15 min at room temperature. Third, 100 uL of different concentrations of LE were added and incubated for 6 h, 12 h, 24 h, 48 h, and 72 hours (as adapted from research conducted by Bachtiar) ^16^. All residues of the test materials ( *E. faecalis* + lime extract) in the microplate were aspirated and the plate was settled for 10 min at room temperature. Then, 50 μL of 2% violet crystalline were added to each well for 5 minutes; the wells were washed with phosphate buffer saline (PBS) two times (Merck, Darmstadt, Germany). A total of 100 μL of Lugol solution was added for 1 minute and washed with PBS. Cell metabolites and dye were removed by the addition of 100 μL of 96% alcohol for 20 seconds. Safranin solution, 50 μL, were added for 2 minutes and washed with PBS ^17, 18^. The microplate was measured using an Elisa reader using with optical density of 595 nm ^13^. For each period, the turbidity of the medium was visually compared with a 0.5 McFarland standard

### Statistical analysis

*E. faecalis* acid tolerance and adhesion to lime extract were calculated to determine average values and standard deviations for each concentration. Two-ways analysis of variance (ANOVA) was performed with significance set at p < 0.05. The analysis was performed using SPSS ver. 20.0 software.

## Results

**Figure 1.**
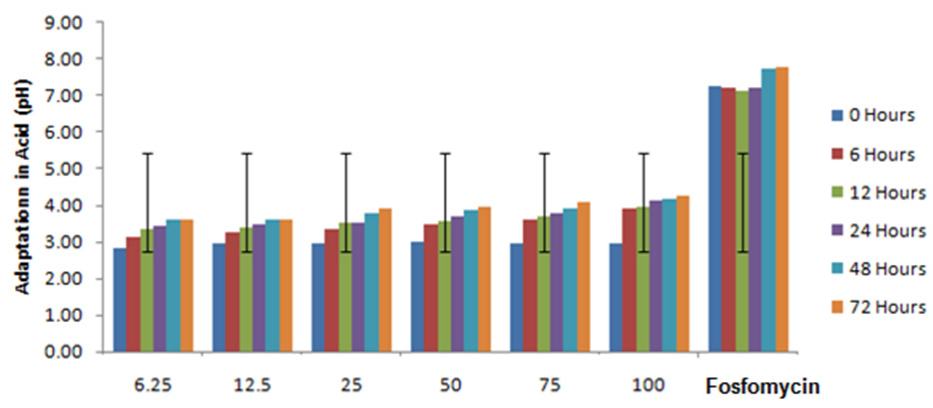
The pH adaptation response of *E. faecalis* to lime extract at different concentration and exposed time.

The experiment was performed in triplicate wells. This study showed that the presence of lime extracts decreased pH, but reduction of low pH did not have a significant effect on the ability of *E. faecalis* to adhere and form biofilm, compared to the control (fosfomycin). All results (adaptation to pH, acid tolerance assays and interaction activity are shown in Figure 1– Figure 3). Interestingly, the tolerance effect was not influenced by exposure time and the concentrations of lime peel extract set in this study ( Figure 3), and the correlation between time exposure and lime extract concentration was positive (r2 = 0.98).

**Figure 2.**
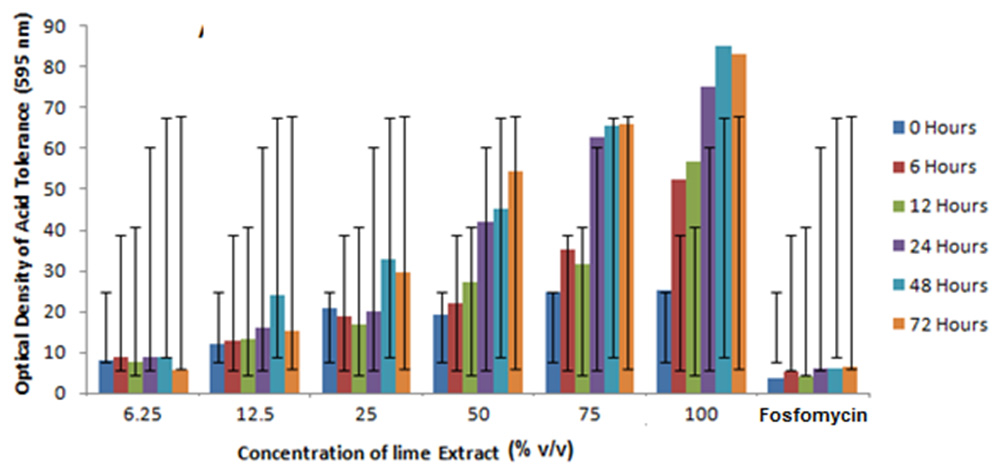
The optical density of the acid tolerance response of *E. faecalis* to lime extract at different concentration and exposed time.

**Figure 3.**
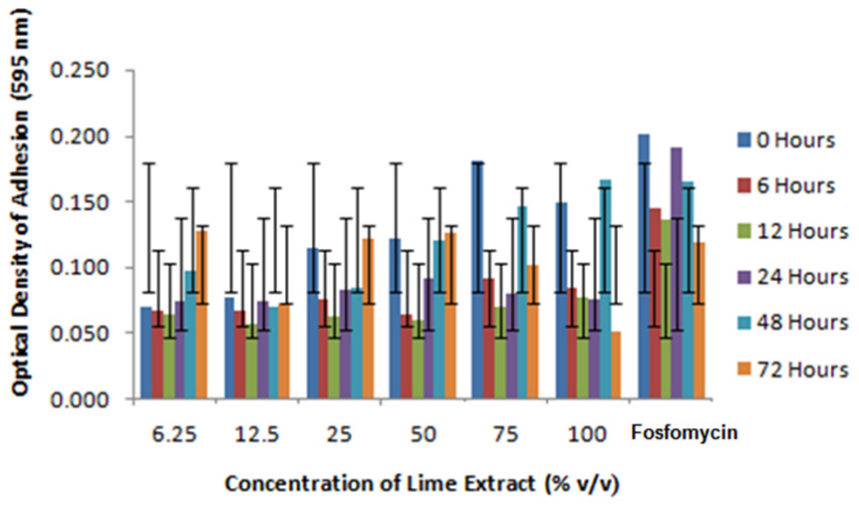
Optical density of adhesion of *E. faecalis* at different concentration of lime extracts and exposed time.

*E. faecalis* did not express an ability to adapt to result in pH changes after interacting with fosfomycin (as a positive control) ( Figure 1), although it still expressed acid tolerance response ( Figure 2) and robust adhesion activity ( Figure 3).

pH adaptation of *E. Faecalis* in lime extract based on replications Click here for additional data file. Copyright: © 2018 Mubarak Z and Soraya C 2018 Data associated with the article are available under the terms of the Creative Commons Zero "No rights reserved" data waiver (CC0 1.0 Public domain dedication).

Optical density (OD) of acid tolerance respond of *E. Faecalis* in lime extract based on replications Click here for additional data file. Copyright: © 2018 Mubarak Z and Soraya C 2018 Data associated with the article are available under the terms of the Creative Commons Zero "No rights reserved" data waiver (CC0 1.0 Public domain dedication).

The OD value of the interaction activity of *E. Faecalis* in lime extract based on replications Click here for additional data file. Copyright: © 2018 Mubarak Z and Soraya C 2018 Data associated with the article are available under the terms of the Creative Commons Zero "No rights reserved" data waiver (CC0 1.0 Public domain dedication).

## Discussion

In searching for pH adaptation of *E. faecalis* to ** ethanolic extract of lime peel, the initial pH of concentrated extract (100% v/v) without *E. faecalis* was 2.89. This is in agreement with those reported by Sitanggang *et al.* ^19^ that the water extract of lime has highly acidic pH ranges (1.7–3.1). As illustrated in Figure 1, this highly acidic pH only slightly changed under serial dilution and after the addition of *E. faecalis* suspension prior to incubation (0 hour).

This relatively stable pH of lime extract after serial dilution in water and after the addition of *E. faecalis* might be related to the presence of buffering compounds in the extract that is able to maintain pH. According to Bolhari *et al.* the juice of lime ( *C. aurantiifolia*) contains 88% water, 6–8% citric acid, 2% potassium citrate and calcium, 0.4–0.6% and other substances ^20^. The water extract of the fruit contains a number of bioactive compounds such as alkaloids, phenols, flavonoids, steroids, terpenoids, reducing sugar, saponins and cardiac glycosides ^21^. Moreover, the peel of *C. aurantiifolia* contained 7 percent essential oil consisting of 46 compounds, most of which are terpenes ^22^. The acidity is generated by citric acid and amino acids, while the essential oils contribute to maintaining its acidic pH ^23^. Citric acid is reported to play a crucial role as a natural material to maintain pH balance and possesses antibacterial activity ^24^.

After incubation there was a significant increase in the acidic pH of lime extract containing *E. faecalis* compared to lime extract alone (as a negative control) (p < 0.05). The increased pH occurred in all different concentration of lime extracts, from 6.25% (v/v) to 100% (v/v) (see Figure 1) indicates that *E. faecalis* can adapt to environments with an acidic pH (2.9–4.2) at a temperature of 37°C. The ability of *E. faecalis* to adapt to situations with a low pH and temperature has been reported by Morandi *et al.* ^25^ in the experiment using pH adjusted to pH of 5.0 at 25°C within 10 hours.

Whilst better *E. faecalis* growth was observed in different lime extract concentrations with long exposure, increased acid tolerance response shown by the microbe as the concentration of the lime extract increased ( Figure 2) (p < 0.05). These phenomena probably relate to the ability of *E. faecalis* to produce a number of virulence factors in the extreme environment, such as the presence of potent antimicrobial agents or highly acidic condition ^26^. Among them is lipoteichoic acids that contribute to biofilm formation, a bacterial community-based resistant mechanism developed by certain bacteria to survive in extremely disadvantages environment ^27^.

Molecularly, the acid tolerance response of *E. faecalis* is influenced by the *EfCitH* gene, which encodes the citrate transporter protein on the surface of the bacterial cell membrane that acts to maintain the balance of the effects of citric acid generated from the environment ^28^. Sarantinopoulos found that enterococcal strains have metabolic potential against the citrate metabolism; this supports their acid tolerance response to environmental influences such as aroma and fermentation products ^29^. In this research, in the presence of fosfomycin with a pH of 7.2 ( Figure 1) *E. faecalis* could still slightly show pH tolerance. The acid tolerance response is related to the ability of certain *E. faecalis* strains to grow in environments with an alkaline pH (9.5–12) within 48–72 hours ^12^.

In general, *E. faecalis* showed progressively decreased adhesion ability in the presence of different concentrations of lime extract within 6 – 24 hours ( Figure 3). The adhesion started to increase after the microbe was exposure to different concentration of lime extract for 24 hours. The relatively high error bar (standard deviation values) obtained indicates such variation might be existed among the *E. faecalis* isolates toward the pH and concentration of lime extracts.

Varoni *et al.* ^30^ reported that anti-adhesion activity between plant polyphenol-rich extract and *Streptococcus mutans* bacteria was at its maximum within 24 hours, while within 6, 7, and 8 hours, the activity was stable but not yet maximal. This is probably caused by the adaptation and tolerant mechanisms developed by *E. faecalis* against bioactive ** compounds ** presence in the extracts. Lime peel extract contains a number of metabolites (phenols, flavonoids, hydrogen peroxide, tannins, alkaloids, and saponins) have various therapeutic properties such as antibacterial, antioxidant, antifungal, analgesic, and anti-inflammatory ^9^.

The mechanism utilized by bacteria to survive heat and low-pH of the environment operate in many different ways. The most successful means of surviving low-pH stress is the complete avoidance of extremely acidic environments. However, none more critical than the sensing of mild acidification to prevent the potentially lethal consequences of the inappropriate production of potentially antigenic proteins. Bacteria that are forewarned by mild acidification can prepare through the induction of a wide range of protective measures. It can alter the composition of the cell membrane, extrude protons, protect macromolecules, alter metabolic pathways, and generate alkaline ^31^.

The lower adhesion activity observed in *E. faecalis* exposed to different concentrations of lime extract compared to that exposed to fosfomycin indicated better antibacterial activity of lime extract on the microbe than that of fosfomycin. Among active ingredients contained in the lime extract are flavonoids (polyethoxylated flavones and flavanones), coumarin, and terpenoids, all of which act as antibacterials ^32^. Extract prepared from peel, fruit and leaves of lime show promising antibacterial activity against some microbes belong to both gram positive bacteria ( *Staphylococcus aureus, S. epidermis* and *E*. *faecalis*) and gram negative bacteria ( *Klebsiella pneumonia* and *Proteus vulgaris*) ^33^.

## Conclusion

*Enterococcus faecalis* can adapt to environments with a pH of 2.9–4.3 generated by lime extracts. In addition *E. faecalis* also expressed a tolerance response to the acidic environment. The interaction activity of *E. faecalis* in different concentrations of lime extract become stable within 6–12 hours at a temperature of 37°C. Therefore, the lime extract can be used to inhibit the *E. faecalis* growth.

### Data availability

Dataset 1: pH adaptation of *E. Faecalis* in lime extract based on replications 10.5256/f1000research.13990.d196643 ^34^.

Dataset 2: Optical density (OD) of acid tolerance respond of *E. Faecalis* in lime extract based on replications 10.5256/f1000research.13990.d196644 ^35^.

Dataset 3: The OD value of the interaction activity of *E. Faecalis* in lime extract based on replications 10.5256/f1000research.13990.d196646 ^36^.
