# Supplementary material for: The acid tolerance response and pH adaptation of Enterococcus faecalis in extract of lime Citrus aurantiifolia from Aceh Indonesia
Source: F1000Res. 2018 Apr 11;7:287. Originally published 2018 Mar 7. [Version 2] doi: 10.12688/f1000research.13990.2 (PMC5897787; doi:10.12688/f1000research.13990.2)
Supplement: pH adaptation of E. Faecalis in lime extract based on replications [file f1000research-7-15751-s0000.tgz › 6cbf33f8-87ae-4739-beef-dd7ba64a94da_dataset_1.docx]

Raw data 1: pH adaptation of *E. Faecalis* in lime extract based on replications

| Concentration | Exposed time | Replication |  |  |  |  |
| --- | --- | --- | --- | --- | --- | --- |
| 6.25 |  | R1 | R2 | R3 | average | SD |
|  | 0 Hours | 2.83 | 2.82 | 2.84 | 2.830 | 0.01 |
|  | 6 Hours | 3.11 | 3.12 | 3.1 | 3.110 | 0.01 |
|  | 12 Hours | 3.1 | 3.3 | 3.68 | 3.360 | 0.29 |
|  | 24 Hours | 3.51 | 3.53 | 3.25 | 3.430 | 0.16 |
|  | 48 Hours | 3.53 | 3.69 | 3.56 | 3.593 | 0.09 |
|  | 72 Hours | 3.62 | 3.63 | 3.61 | 3.620 | 0.01 |
| 12.5 |  |  |  |  |  |  |
|  | 0 Hours | 2.98 | 2.96 | 2.97 | 2.97 | 0.01 |
|  | 6 Hours | 3.24 | 3.32 | 3.22 | 3.26 | 0.05 |
|  | 12 Hours | 3.35 | 3.32 | 3.44 | 3.37 | 0.06 |
|  | 24 Hours | 3.5 | 3.46 | 3.42 | 3.46 | 0.04 |
|  | 48 Hours | 3.58 | 3.6 | 3.59 | 3.59 | 0.01 |
|  | 72 Hours | 3.64 | 3.63 | 3.62 | 3.63 | 0.01 |
| 25 |  |  |  |  |  |  |
|  | 0 Hours | 2.98 | 2.95 | 2.98 | 2.97 | 0.02 |
|  | 6 Hours | 3.4 | 3.3 | 3.4 | 3.37 | 0.06 |
|  | 12 Hours | 3.5 | 3.58 | 3.52 | 3.53 | 0.04 |
|  | 24 Hours | 3.58 | 3.5 | 3.52 | 3.53 | 0.04 |
|  | 48 Hours | 3.73 | 3.69 | 3.9 | 3.77 | 0.11 |
|  | 72 Hours | 3.85 | 3.93 | 3.98 | 3.92 | 0.07 |
| 50 |  |  |  |  |  |  |
|  | 0 Hours | 3.02 | 2.95 | 2.99 | 2.99 | 0.04 |
|  | 6 Hours | 3.54 | 3.28 | 3.56 | 3.46 | 0.16 |
|  | 12 Hours | 3.51 | 3.5 | 3.68 | 3.56 | 0.10 |
|  | 24 Hours | 3.73 | 3.75 | 3.58 | 3.69 | 0.09 |
|  | 48 Hours | 3.74 | 3.9 | 3.98 | 3.873 | 0.12 |
|  | 72 Hours | 3.92 | 3.97 | 3.97 | 3.95 | 0.03 |
| 75 |  |  |  |  |  |  |
|  | 0 Hours | 2.94 | 2.98 | 2.97 | 2.96 | 0.02 |
|  | 6 Hours | 3.28 | 3.59 | 3.94 | 3.60 | 0.33 |
|  | 12 Hours | 3.67 | 3.75 | 3.62 | 3.68 | 0.07 |
|  | 24 Hours | 3.71 | 3.73 | 3.88 | 3.77 | 0.09 |
|  | 48 Hours | 3.94 | 3.91 | 3.92 | 3.923 | 0.02 |
|  | 72 Hours | 3.99 | 4.08 | 4.13 | 4.067 | 0.07 |
| 100 |  |  |  |  |  |  |
|  | 0 Hours | 2.95 | 2.97 | 2.99 | 2.97 | 0.02 |
|  | 6 Hours | 3.93 | 3.84 | 3.94 | 3.90 | 0.06 |
|  | 12 Hours | 3.9 | 4.17 | 3.85 | 3.97 | 0.17 |
|  | 24 Hours | 3.9 | 4.19 | 4.29 | 4.13 | 0.20 |
|  | 48 Hours | 4.3 | 4.22 | 4.045 | 4.188 | 0.13 |
|  | 72 Hours | 4.24 | 4.2 | 4.34 | 4.26 | 0.07 |
| Fosfomicin |  |  |  |  |  |  |
|  | 0 Hours | 7.33 | 7.27 | 7.19 | 7.26 | 0.07 |
|  | 6 Hours | 7.09 | 7.27 | 7.34 | 7.23 | 0.13 |
|  | 12 Hours | 7.12 | 7.2 | 7.07 | 7.13 | 0.07 |
|  | 24 Hours | 7.16 | 7.24 | 7.29 | 7.23 | 0.07 |
|  | 48 Hours | 7.84 | 7.71 | 7.68 | 7.74 | 0.09 |
|  | 72 Hours | 7.68 | 7.96 | 7.71 | 7.78 | 0.15 |
