# Supplementary material for: The acid tolerance response and pH adaptation of Enterococcus faecalis in extract of lime Citrus aurantiifolia from Aceh Indonesia
Source: F1000Res. 2018 Apr 11;7:287. Originally published 2018 Mar 7. [Version 2] doi: 10.12688/f1000research.13990.2 (PMC5897787; doi:10.12688/f1000research.13990.2)
Supplement: Optical density (OD) of acid tolerance respond of E. Faecalis in lime extract based on replications [file f1000research-7-15751-s0001.tgz › 7854c956-f250-43ed-b341-af566ca5e95f_dataset_2.docx]

Raw data 2: Optical density (OD) of acid tolerance respond of E. Faecalis in lime extract based on replications

| Concent | Exposed time | Replication | | | Mean | SD |
| --- | --- | --- | --- | --- | --- | --- |
| 6.25 |  | U1 | U2 | U3 |  |  |
|  | 0 Hours | 8 | 8 | 8 | 8.0 | 0.0 |
|  | 6 Hours | 8 | 8 | 10 | 8.7 | 1.2 |
|  | 12 Hours | 8 | 7 | 8 | 7.7 | 0.6 |
|  | 24 Hours | 9 | 9 | 9 | 9.0 | 0.0 |
|  | 48 Hours | 10 | 8 | 9 | 9.0 | 1.0 |
|  | 72 Hours | 4 | 5 | 8 | 5.7 | 2.1 |
| 12.5 |  |  |  |  |  |  |
|  | 0 Hours | 11 | 14 | 11 | 12 | 1.7 |
|  | 6 Hours | 14 | 14 | 11 | 13 | 1.7 |
|  | 12 Hours | 13 | 13 | 14 | 13 | 0.6 |
|  | 24 Hours | 17 | 14 | 17 | 16 | 1.7 |
|  | 48 Hours | 26 | 22 | 24 | 24 | 2.0 |
|  | 72 Hours | 13 | 15 | 17 | 15 | 2.0 |
| 25 |  |  |  |  |  |  |
|  | 0 Hours | 22 | 21 | 20 | 21.0 | 1.0 |
|  | 6 Hours | 19 | 19 | 18 | 18.7 | 0.6 |
|  | 12 Hours | 17 | 17 | 16 | 16.7 | 0.6 |
|  | 24 Hours | 20 | 21 | 19 | 20.0 | 1.0 |
|  | 48 Hours | 33 | 33 | 32 | 32.7 | 0.6 |
|  | 72 Hours | 27 | 31 | 31 | 29.7 | 2.3 |
| 50 |  |  |  |  |  |  |
|  | 0 Hours | 22 | 20 | 16 | 19 | 3.1 |
|  | 6 Hours | 21 | 21 | 24 | 22 | 1.7 |
|  | 12 Hours | 27 | 31 | 24 | 27 | 3.5 |
|  | 24 Hours | 41 | 40 | 45 | 42 | 2.6 |
|  | 48 Hours | 46 | 43 | 47 | 45 | 2.1 |
|  | 72 Hours | 50 | 58 | 55 | 54 | 4.0 |
| 75 |  |  |  |  |  |  |
|  | 0 Hours | 25 | 25 | 24 | 24.7 | 0.6 |
|  | 6 Hours | 36 | 36 | 33 | 35.0 | 1.7 |
|  | 12 Hours | 32 | 30 | 33 | 31.7 | 1.5 |
|  | 24 Hours | 65 | 63 | 61 | 63.0 | 2.0 |
|  | 48 Hours | 66 | 66 | 65 | 65.7 | 0.6 |
|  | 72 Hours | 62 | 68 | 68 | 66.0 | 3.5 |
| 100 |  |  |  |  |  |  |
|  | 0 Hours | 26 | 25 | 25 | 25 | 0.6 |
|  | 6 Hours | 54 | 51 | 52 | 52 | 1.5 |
|  | 12 Hours | 59 | 58 | 54 | 57 | 2.6 |
|  | 24 Hours | 78 | 71 | 77 | 75 | 3.8 |
|  | 48 Hours | 85 | 88 | 83 | 85 | 2.5 |
|  | 72 Hours | 84 | 85 | 81 | 83 | 2.1 |
| Fosfomicin |  |  |  |  |  |  |
|  | 0 Hours | 3 | 4 | 4 | 4 | 0.6 |
|  | 6 Hours | 5 | 5 | 6 | 5 | 0.6 |
|  | 12 Hours | 4 | 4 | 4 | 4 | 0.0 |
|  | 24 Hours | 6 | 7 | 5 | 6 | 1.0 |
|  | 48 Hours | 6 | 6 | 6 | 6 | 0.0 |
|  | 72 Hours | 6 | 7 | 6 | 6 | 0.6 |
