# Supplementary material for: The acid tolerance response and pH adaptation of Enterococcus faecalis in extract of lime Citrus aurantiifolia from Aceh Indonesia
Source: F1000Res. 2018 Apr 11;7:287. Originally published 2018 Mar 7. [Version 2] doi: 10.12688/f1000research.13990.2 (PMC5897787; doi:10.12688/f1000research.13990.2)
Supplement: The OD value of the interaction activity of E. Faecalis in lime extract based on replications [file f1000research-7-15751-s0002.tgz › a1bc94c2-9d51-4d94-ade3-33ffe57541c8_dataset_3.docx]

Raw data 3: The OD value of the interaction activity of *E. Faecalis* in lime extract based on replications

| Concent | Exposed time | Replication | | | Average | SD |
| --- | --- | --- | --- | --- | --- | --- |
| 6.25 |  | R1 | R2 | R3 |  |  |
|  | 0 Hours | 0.06 | 0.07 | 0.08 | 0.075 | 0.007 |
|  | 6 Hours | 0.06 | 0.07 | 0.07 | 0.070 | 0.000 |
|  | 12 Hours | 0.06 | 0.06 | 0.07 | 0.065 | 0.007 |
|  | 24 Hours | 0.07 | 0.07 | 0.08 | 0.075 | 0.007 |
|  | 48 Hours | 0.09 | 0.1 | 0.1 | 0.100 | 0.000 |
|  | 72 Hours | 0.12 | 0.13 | 0.13 | 0.130 | 0.000 |
| 12.5 |  |  |  |  |  |  |
|  | 0 Hours | 0.07 | 0.08 | 0.08 | 0.077 | 0.006 |
|  | 6 Hours | 0.03 | 0.1 | 0.07 | 0.067 | 0.035 |
|  | 12 Hours | 0.06 | 0.04 | 0.07 | 0.057 | 0.015 |
|  | 24 Hours | 0.06 | 0.07 | 0.09 | 0.073 | 0.015 |
|  | 48 Hours | 0.07 | 0.09 | 0.05 | 0.070 | 0.020 |
|  | 72 Hours | 0.06 | 0.095 | 0.063 | 0.073 | 0.019 |
| 25 |  |  |  |  |  |  |
|  | 0 Hours | 0.088 | 0.15 | 0.106 | 0.115 | 0.032 |
|  | 6 Hours | 0.068 | 0.075 | 0.083 | 0.075 | 0.008 |
|  | 12 Hours | 0.06 | 0.06 | 0.065 | 0.062 | 0.003 |
|  | 24 Hours | 0.08 | 0.094 | 0.074 | 0.083 | 0.010 |
|  | 48 Hours | 0.089 | 0.079 | 0.085 | 0.084 | 0.005 |
|  | 72 Hours | 0.127 | 0.122 | 0.115 | 0.121 | 0.006 |
| 50 |  |  |  |  |  |  |
|  | 0 Hours | 0.128 | 0.088 | 0.148 | 0.121 | 0.031 |
|  | 6 Hours | 0.072 | 0.058 | 0.06 | 0.063 | 0.008 |
|  | 12 Hours | 0.06 | 0.065 | 0.053 | 0.059 | 0.006 |
|  | 24 Hours | 0.086 | 0.064 | 0.122 | 0.091 | 0.029 |
|  | 48 Hours | 0.122 | 0.123 | 0.114 | 0.120 | 0.005 |
|  | 72 Hours | 0.134 | 0.123 | 0.119 | 0.125 | 0.008 |
| 75 |  |  |  |  |  |  |
|  | 0 Hours | 0.191 | 0.181 | 0.17 | 0.181 | 0.011 |
|  | 6 Hours | 0.09 | 0.098 | 0.083 | 0.090 | 0.008 |
|  | 12 Hours | 0.077 | 0.061 | 0.072 | 0.070 | 0.008 |
|  | 24 Hours | 0.1 | 0.082 | 0.076 | 0.080 | 0.003 |
|  | 48 Hours | 0.147 | 0.119 | 0.17 | 0.145 | 0.026 |
|  | 72 Hours | 0.11 | 0.098 | 0.096 | 0.101 | 0.008 |
| 100 |  |  |  |  |  |  |
|  | 0 Hours | 0.114 | 0.188 | 0.146 | 0.149 | 0.037 |
|  | 6 Hours | 0.073 | 0.088 | 0.092 | 0.084 | 0.010 |
|  | 12 Hours | 0.075 | 0.083 | 0.07 | 0.076 | 0.007 |
|  | 24 Hours | 0.073 | 0.072 | 0.082 | 0.076 | 0.006 |
|  | 48 Hours | 0.134 | 0.178 | 0.188 | 0.167 | 0.029 |
|  | 72 Hours | 0.052 | 0.053 | 0.048 | 0.051 | 0.003 |
| Fosfomicyn |  |  |  |  |  |  |
|  | 0 Hours | 0.25 | 0.193 | 0.158 | 0.200 | 0.046 |
|  | 6 Hours | 0.186 | 0.109 | 0.14 | 0.145 | 0.039 |
|  | 12 Hours | 0.162 | 0.15 | 0.095 | 0.136 | 0.036 |
|  | 24 Hours | 0.18 | 0.215 | 0.176 | 0.190 | 0.021 |
|  | 48 Hours | 0.201 | 0.125 | 0.17 | 0.165 | 0.038 |
|  | 72 Hours | 0.116 | 0.121 | 0.117 | 0.118 | 0.003 |
