# Supplementary material for: The acid tolerance response and pH adaptation of Enterococcus faecalis in extract of lime Citrus aurantiifolia from Aceh Indonesia
Source: F1000Res. 2018 Apr 11;7:287. Originally published 2018 Mar 7. [Version 2] doi: 10.12688/f1000research.13990.2 (PMC5897787; doi:10.12688/f1000research.13990.2)
Supplement: Peer review history

# Peer review: Referee response for version 2

**DOI:** 10.5256/f1000research.15751.r33038

**Published:** 2018-04-12

**Authors:** Boy M. Bachtiar

**Affiliations:** 1 Department of Oral Biology and Oral Science Research Center, Faculty of Dentistry, Universitas Indonesia, Jakarta, Indonesia

I have read the revised manuscript, and the authors have made modifications to the manuscript, and have no further comments. Thus, I recommend this work for publication in F1000Research.

 Thank you for considering me as a reviewer.

 Kindly Regards

 Boy M Bachtiar

I have read this submission. I believe that I have an appropriate level of expertise to confirm that it is of an acceptable scientific standard.

# Peer review: Referee response for version 1

**DOI:** 10.5256/f1000research.15206.r31610

**Published:** 2018-03-19

**Authors:** Elza Ibrahim Auerkari

**Affiliations:** 1 Department of Oral Biology, Faculty of Dentistry, Universitas Indonesia, Jakarta, Indonesia

In general, the paper would still benefit from a review by a native English speaker to correct the language.

 Additional recommended actions to consider are the following:

**Abstract**

 in Methods (2nd line): instead of “Both materials were analyzed for their adaptation, …” please use “The microbe was analyzed for its adaptation, …”  

**Methods**

 in Culture of *E. faecalis *bacteria: please rephrase the last sentence “The Accurate of the density ...” to make it more understandable.

 in Adaptation to pH assay: first sentence: how do the concentration values, given here in %, compare with units of Figures 1 to 3 where concentration is given in micrograms per milliliter?

**Results, Discussion**

 Figure 1: why is the initial pH value (at 0 hours) about the same at all lime extract concentrations? Are there any known inhibitor (buffering) substances in the lime extract (or in the bacterial culture) that could explain relatively constant initial pH at a range of concentrations?

 Figure 2: why is the indicated acid tolerance apparently higher towards increasing lime extract concentration also at 0 hours of exposure?

 Figure 3: why is the indicated adhesion level apparently higher towards increasing lime extract concentration also at 0 hours of exposure? Where are the error bars mentioned in the text?

 The paper is referring in many places to “interaction activity” between lime extract and *E. faecialis*. However, could the results also involve a concentration-dependent interaction between e.g. citric acid and other antibacterial compounds of the extract?

I have read this submission. I believe that I have an appropriate level of expertise to confirm that it is of an acceptable scientific standard.

# Response

**Published:** 2018-03-29

**Authors:** Zaki Mubarak

**Affiliations:** Syiah Kuala University, Indonesia

We have made the revision based on suggestion and comment from reviewers

# Peer review: Referee response for version 1

**DOI:** 10.5256/f1000research.15206.r31653

**Published:** 2018-03-15

**Authors:** Boy M. Bachtiar

**Affiliations:** 1 Department of Oral Biology and Oral Science Research Center, Faculty of Dentistry, Universitas Indonesia, Jakarta, Indonesia

**Introduction**

 The last paragraph: ………..acid tolerance response and pH adaptation of E. faecalis  when it interacts with lime extract.   

 Reviewer’s comment:  please change the bold word with: when the bacterium grows as biofilm in the presence of lime extract with difference concentration.

**Method**

 -           “Both materials were made in vitro to analyze”.

 Reviewer’s comment: please change with: The material and bacterium were prepared

 - In lime extract, typo……………………………………into a glass

 - Culture of E. faecalis bacteria; please discard; “bacteria”

 - E. faecalis ATCC 29212 taken from glycerol ……………………………………………………………………………………………..and incubator (Memmert, Germany). 

 Reviewer’s suggestion: discard this part, and please replace with:

 One colony of E. faecalis bacteria was subsequently re-cultured in 5 ml of Mueller-Hinton Broth (MHB) medium (Thermo Fisher Scientific Inc, Paisley, UK) in anaerobic conditions at a temperature of 37°C for 48 hours. Afterward, the E. faecalis grown on the liquid medium was synchronized further with McFarland 0.5 (1 x 108 CFU/ml) (TM50, Dalynn Biological Inc., Calgary, Canada). The Accurate of the density of McFarland standart (typo: it should be standard) can be checked using a spectrophotuometer with an absorbance reading of 0.08 to 0.1 at 625 nm11.

 Adaptation to pH assay

 A total of 50 ml of lime extracts in several different concentrations

 (100%, 75%, 50%, 25%, 12.5%, and 6.25%) was placed into different beaker glasses. Then, 5 ml containing  1 X 108 CFU/mL of E. faecalis in MHB (1:10) were added to each of the beakers. The initial pH

 Reviewer’s suggestion: please indicate the initial pH that was measured at the zero h.,  before incubation. Reviewer’s suggestion:  Next, bacterium-containing beaker was put into incubator (37°C)  for 6 hours, 12, hours, 24 hours, 48 hours, and 72 hours in an anaerobic atmosphere using Anaerogen TM GasPack (Oxoid, Basing stoke UK), at each of these times, the beakers’ pH was measured using a pH meter (Thermo Fisher Scientific Inc, Paisley, UK). Various changes in pH from 0 hours to the specified time can be used as an indicator of whether E. faecalis has a tolerance response to the acidic environment and can adapt to changing pH.

 Acid tolerance assay

 -           “Here, 96 wells of the triple microplate series were coated with 50 μl of MHB”.

 Reviewer suggestion: Please change to: Here, 50 ul of MHB was put into microplate in triplicate.

 -           “the test materials (E. faecalis + lime extract) derived…….

 Reviewer suggestion: please change to: material tested  and E. faecalis……………………………………………”Each well” change to the microplate was put into incubator for 15 min.

 -           “Then bio-tolerant activity was measured using the Elisa Reader (Bio-Rad Laboratories, Hercules, CA) at a wavelength of 595 nm”. I suggest to discard this part, as it does not has a clear meaning, ….activity was measure by ELISA reader……..

 Adhesion assay

 -           “The standard protein concentration of the E. faecalis and the active component concentration of the lime extract were measured via the Bradford method”

 It is not clear, why the authors need to analyze (by measuring) the E. faecalis’s proteins (I assume the whole cell proteins). Thus, what does the authors would like to say with "the standard protein concentration of E. faecalis and the concentration of active component of the lime extract”?. My suggestion is please discard this part.

 -           “Spectrophotometry detected the interaction of E. faecalis with lime extract at a wavelength of 595 nm”

 It is rather confusing. Spectrophotometry was used to detect the interaction between the bacterium and the lime extract. Did  you use this method to determine the bacterium growth rate? I suggest to discard this spectrometric method. Otherwise, please focus the methods (adaptation to pH, acid tolerance, and adhesion assay) used only to address your hypothesis; "that biofilm formation or bacterial adherence ability contributes to E. faecalis survival in biofilm-related environment in the presence of lime extract."

 -           The principle of incubation time-based interaction activity on the microplate 96 wells series used in this research based on Gamble’s working principle. I think you only tested the bacterial adherence capability, not interaction activity between E. faecalis and……….?

 -           It was modified using violet crystalline and safranin staining. First, 96 wells of the triple microplate series were coated with………..

 Suggestion: Please change this part: “First, microplate in triplicate wells. ...................Second, 50 uL of E. faecalis in ( ADD IN WHAT MEDIUM USED) ...........Third, 100 uL of different concentration of LE was added and incubated for..........

 -           “A total of 100 μL of Lugol solution was added for 1 minute and then washed with PBS. The rest of the cell metabolism that was not bacterial cells was dissolved in 96% alcohol for 20 seconds until the dye completely removed. 50 μL of safranin solution was added for 2 minutes and then washed again with PBS18. The interaction activity between the lime extract and the E. faecalis bacteria in the microplate wells was assessed via an Elisa reader using a spectrophotometer (Bio-Rad Laboratories, Hercules, CA, USA) at a wavelength of 595 nm”

 Suggestion:

1. As you only tested mono-species biofilm (E. faecalis is a Gram (+) ve bacterium), please add an explanation, why you used both crystal violet and safranin solution?, and you did it similar to the procedure for Gram staining method. 
2. Please discard the word: “The interaction activity between the lime extract and the E. faecalis Bacteria”. It would better to say: the bacterial adherence was assessed using crystal violet and measured using ELISA reader with optical density of 595 nm.
3. Observation of adhesion mass. This method is not necessary for this study, as you have used crystal violet (CV) assay to measure biofilm mass. CV provides a good measure of biofilm mass, although it cannot measure biofilm viability.  This means, CV stains both  bacteria cells and the polymer substance (carbohydrate) as part of the extra cellular matrix.

 

**Statistical analysis**

 ………..standard deviations for each concentration.

 Reviewer comment: why there are no SD displayed in each graph shown in the figures

**Results**

**-**The experiment was conducted in three replicates

 Reviewer suggestion:

  

1. Please changed:  the experiment was performed in triplicate wells
2. This study showed that the presence of lime extracts decreased pH, but reduction of low pH did not have a significant effect on the ability of E. faecalis to adhere and form biofilm, compared to the control (fosfomycin). All result (crystal violet, adaptation to pH, and acid tolerance assays) are shown in Fig .1-3).
3. Interestingly, the tolerance effect was not influenced by exposure time and the concentration of LE set in this study (Fig. 3), and the correlation between time exposure and LE concentration was positive (However, the authors need to show the regression graph as they said r2 = 0.98). Otherwise, please exclude this part.
4. “the results showed that the interaction activities of E. faecalis in Lime extract will decrease from 6, 12, 24, and 72 hours. Reviewer‘s comment: I am wondering what the authors mean with the interaction activity. I suggest to discard this part.
5. The mass profile also indicated interaction activity between lime extract and E. faecalis. Antibiotics are capable of forming a covalent bond to activate the cysteine residue of a bacterial cell, triggering UDP-N-acetylglucosamine to form hydrogen bonds. It inhibits the synthesis of peptidoglycan as an antibacterial defense. Reviewer’s comment: Please discard this part, as 1. I cannot see the data (mass profile). 2. Antibiotic are capable........this is not the result of this study. When necessary please put the sentence in the discussion section.

 

**Discussion**

 Reviewer’s comment: please focus the results you got. Especially, please do not repeat the result, and exclude: “The interaction activity between lime extract and E. faecalis can be assumed to be the antibacterial activity, because there was a decrease in the interaction activity between E. faecalis and the biological components of lime extract within 6–24 hours and again within 48–72 hours. The interaction activity is related to the activity of the active ingredients contained in the lime extract, such as flavonoids (polyethoxylated flavones and flavanones), coumarin, and terpenoids, all of which act as antibacterials”. I suggest to rewrite this part ……….The survival of E. faecalis in the presence of LE………..

**Conclusion**

 E. faecalis can adapt to environments with a pH of 2.9–4.3 generated by lime extracts. In addition E. faecalis also expressed a tolerance response to the acidic environment. The interaction activity between E. faecalis and lime extract become stable within 6–12 hours at a temperature of 37°C. Therefore, the lime extract can be used to inhibit the E. faecalis growth.

 Reviewer’s comment: Your conclusion should succinctly describe the overall result of the experiment. Please delete: 1. The interaction activity between E. faecalis and lime extract become stable within 6–12 hours at a temperature of 37°C. 2/.  Therefore, the lime extract can be used to inhibit the E. faecalis growth.

 Please refer your conclusion to the title that reflects the result.

I have read this submission. I believe that I have an appropriate level of expertise to confirm that it is of an acceptable scientific standard, however I have significant reservations, as outlined above.

# Response

**Published:** 2018-03-29

**Authors:** Zaki Mubarak

**Affiliations:** Syiah Kuala University, Indonesia

Revision has been made according to the comment of the reviewer
